# Differential Susceptibility to Cadmium-Induced Liver and Kidney Injury in Wild and Laboratory-Bred Bank Voles *Myodes glareolus*

**DOI:** 10.1007/s00244-013-9896-2

**Published:** 2013-04-06

**Authors:** Aneta Salińska, Tadeusz Włostowski, Ewa Oleńska

**Affiliations:** Institute of Biology, University of Białystok, Świerkowa 20B, 15-950 Białystok, Poland

## Abstract

The objective of the study was to compare the sensitivity of wild and laboratory-bred bank voles to cadmium (Cd)-induced histopathological changes in the liver and kidneys. For 4 weeks, the male bank voles—both wild and laboratory-bred—were provided with diet containing Cd in quantities <0.1 (control), 30, and 60 μg/g dry weight. At the end of exposure period, histopathology and analyses of Cd, metallothionein (MT), glutathione (GSH), zinc (Zn), copper (Cu), iron (Fe), and lipid peroxidation—all considered to be critical factors during the development of Cd toxicity in the liver and kidneys—were carried out. Histopathological changes (focal hepatocyte swelling, vacuolation and inflammation [leukocyte infiltration] in the liver, and focal proximal tubule degeneration [including epithelial cell swelling] in the kidneys) occurred only in the wild bank voles fed a diet containing 60 μg Cd/g. There were no differences in concentrations of Cd, MT, GSH, Zn, and Cu in liver and kidney between the respective groups of wild and laboratory-bred animals. However, a decrease of hepatic Fe and lipid peroxidation was observed in the wild voles exhibiting histopathological changes. These data indicate the following: (1) wild bank voles are more susceptible to Cd-induced liver and kidney injury than those bred and raised in the laboratory; (2) the difference in sensitivity may be associated with a distinct decrease of hepatic Fe in response to Cd exposure between the two groups of bank voles; and (3) dietary Cd may produce histopathological changes indirectly through decreasing the hepatic Fe and Fe-dependent oxidative processes. These results also suggest that histopathology in the liver and kidney of wild bank voles living in a contaminated environment may occur at relatively low levels of tissue Cd.

Cadmium (Cd) is a toxic metal widely distributed in the environment as a result of industrial and agricultural practices (Liu 2003; Satarug et al. 2003; Thévenod 2009). The source of Cd intake is mostly food, and most of the metal that is absorbed after oral exposure mainly accumulates in the liver and kidney (Lehman and Klaassen 1986), where it induces production of metallothionein (MT), a low molecular–weight protein that binds Cd with high affinity (Klaassen et al. 2009). The protein forming a complex with Cd decreases its free concentration within the cell, thus decreasing the toxic potential of Cd. When the binding capacity of MT becomes saturated, the increased level of unbound Cd ions initiates processes that can lead to liver and kidney injury (Goyer et al. 1989; Włostowski et al. 2004). In liver, chronic Cd exposure produces nonspecific inflammation, hepatocyte swelling, and mild necrosis (Habeebu et al. 2000; Włostowski et al. 2000, 2004). The following processes may be involved in the development of hepatotoxicity: (1) Cd activation of Kupffer cells that induce inflammation (Kuester et al. 2002); (2) Cd injury of hepatic endothelial cells that obstruct the capillary lumen, thereby producing local hypoxia (Liu et al. 1992; Kuester et al. 2002); and (3) Cd induction of hepatic iron (Fe) depression, which may cause disturbances in Fe-dependent oxidative processes, *e.g.*, adenosine triphosphate (ATP) synthesis (Włostowski et al. 2000, 2003). It is thought that injured hepatocytes release a Cd–MT complex into the blood, which is then filtered in the kidneys through glomeruli and reabsorbed by proximal tubule epithelial cells, which are the target for extracellular Cd–MT (Sabolić et al. 2010). After degradation of the complex in these cells, Cd ions induce oxidative stress that causes, among others, mitochondrial swelling and loss of cristae as well as an inhibition of Na^+^–K^+^-ATPase (Thévenod and Friedmann 1999; Wang et al. 2010). These events eventually lead to loss of ionic control and cellular injury.

During chronic exposure, Cd-induced toxicity in liver and kidney is dependent on hepatic and renal Cd concentrations (Groten et al. 1994; Liu et al. 1998; Włostowski et al. 2004). Notably, various wild animals inhabiting polluted sites exhibit histopathological changes in liver or kidney at Cd concentrations that are several-fold lower compared with those in animals exposed to Cd under laboratory conditions (Beiglböck et al. 2002; Damek-Poprawa and Sawicka-Kapusta 2004; Sanchez-Chardi et al. 2009; Włostowski et al. 2010). For example, in a small rodent, the bank vole from an industrialized area, hepatic and renal injury occurs at tissue Cd levels <10 μg/g wet weight (Damek-Poprawa and Sawicka-Kapusta 2004), whereas in the laboratory-bred bank voles exposed to Cd, histopathology in the liver and kidney is evident only when the concentration is >40 μg/g wet weight (Włostowski et al. 2004). However, the exact reason for this difference is not known; to date, no direct comparison of the sensitivity of wild with that of laboratory-bred bank voles and other species exposed to Cd has been reported. Therefore, the objective of the present study was to compare the susceptibility of wild bank voles living in a relatively uncontaminated area with that of laboratory-bred voles to liver and kidney injury induced by dietary Cd. Toxicity was evaluated by assessing liver and kidney histopathology. Analyses of MT, glutathione (GSH), and zinc (Zn), which are known to decrease Cd toxicity (Chan and Cherian 1992; Jacquillet et al. 2006; Jihen et al. 2008), as well as Cd, Fe, copper (Cu) and lipid peroxidation, which are responsible for the progression of toxicity (Thévenod and Friedmann 1999; Liu et al. 2009; Whittaker et al. 2011), were carried out to determine whether the different susceptibility (if any) is associated with MT induction, GSH content, trace-element concentrations, or oxidative stress.

## Materials and Methods

### Animals and Experimental Design

All experimental procedures were approved by the Local Ethical Committee (Medical University of Białystok) and were compatible with the standards of the Polish Law on Experimenting on Animals, which implements the European Communities Council Directive (86/609/EEC). Wild male bank voles (weight 13.5–15.0 g, age 1.5–2.0 months) were caught in September 2011 in live traps in the Knyszyn Old Forest (northeastern Poland, the least contaminated region of Poland). The group of laboratory-bred male bank voles (weight 13–14.5 g, age 1.5 months) were the F2 offspring of the wild-caught stock (Knyszyn Old Forest). The wild and laboratory-bred bank voles were randomly divided into three subgroups (*n* = 6 each) according to dietary Cd exposure: (1) control, (2) Cd-30 μg/g, and (3) Cd-60 μg/g dry weight. The animals were housed individually for 4 weeks in stainless-steel cages (44 × 27 × 20 cm) (lined with peat as absorptive material) at 18–20 °C under a 12:12 hour light–to–dark cycle and at 50–70 % relative humidity. They received *ad libitum* distilled water and control or Cd-containing whole wheat grains, which appeared to be an adequate-quality food for these rodents (Włostowski et al. 2004). In addition, an identical quantity of apple was offered to all voles (3 g/vole/wk), who ate it completely. Food intake was monitored throughout the experiment. Before the experiment, the grains were contaminated with Cd (soaked in CdCl_2_ solution). Atomic absorption spectrophotometry (AAS) analysis of the grains revealed that actual levels of Cd were between 28 and 33 μg/g (Cd-30) and 58 and 63 μg/g (Cd-60). Control grains contained <0.1 μg Cd/g. The concentrations of Zn, Cu, and Fe in the grains were 22–26, 4–6, and 80–100 μg/g dry weight, respectively. The chosen concentrations of dietary Cd were similar or twofold greater than those found in a heavily contaminated environment (Liu 2003).

At the end of the 4 week exposure period, the bank voles were weighed, killed by decapitation, and the liver and kidneys removed, rinsed in cold saline, and blotted dry on absorbent paper. Blood was also taken to determine hemoglobin and hematocrit by using standard methods (spectrophotometrically as cyanmethemoglobin at 540 nm and hematocrit centrifuge, respectively). A portion of fresh liver (0.25 g) and one kidney were transferred to 1.0 mL of chilled 0.25 M sucrose and homogenized with a Teflon pestle in a glass homogenizer. Aliquots (0.5 and 0.1 mL) of the homogenate were taken for determination of metal concentrations and lipid peroxidation, respectively. The remaining homogenate was centrifuged at 20,000×*g* for 20 min at 4 °C and the resulting supernatant removed for MT and GSH assays.

### Histological Examination

A portion of liver and one kidney were fixed in 4 % formaldehyde, dehydrated in ethanol and xylene, embedded in paraffin, cut into 5 μm sections, and stained with hematoxylin and eosin for microscopic examination.

### Metal Determination

Metal determinations were performed as described recently in Salińska et al. (2012). The homogenate (0.5 mL) was placed in a glass tube with 2.0 mL of concentrated nitric acid (Sigma-Aldrich). After 20 h of sample digestion at room temperature, 72 % perchloric acid (0.5 mL) (Sigma-Aldrich) was added and the mixture heated at 100 °C for 3 h. Finally, the temperature was increased to 150°–180 °C and digestion continued for another 2 h. Deionized water was added to the residue (0.1 mL) after digestion to a volume of 3.0 mL (first solution). A portion of the first solution (200 μL) was evaporated to dryness in a quartz crucible at 130 °C, and the residue was redissolved in an appropriate amount of deionized water (second solution). Cd analyses of these solutions were carried out by electrothermal AAS using a Solaar M6 instrument with a Zeeman correction. The concentrations of Zn, Cu, and Fe in the first solution were determined by AAS in an air-acetylene flame with deuterium correction. Quality-assurance procedures included the analysis of reagent blanks and appropriate standard reference material (National Institute of Standards and Technology bovine liver 1577b). The recoveries of Cd, Zn, Cu, and Fe were 91–93, 90–95, 89–95, and 95–101 %, respectively.

### MT Determination

MT in liver and kidney was determined by Cd-saturation method (Włostowski et al. 2004). Briefly, a 0.1 mL sample was incubated in a 1.5 mL vial for 10 min at room temperature with 1.0 mL Tris-HCl buffer (0.03 M, pH 7.8) containing 1.0 μg Cd/mL. To remove non–MT-bound Cd, bovine hemoglobin (Sigma) (0.1 mL of 5 % solution in H_2_O) was added and the sample heated for 1.5 min at 95 °C, cooled, and centrifuged for 5 min at 10,000×*g*. Addition of hemoglobin, heating, and sample centrifugation was repeated twice. Cd bound to MT in the resulting clear supernatant was determined by electrothermal AAS. MT content was expressed in micrograms of the protein per gram of wet tissue assuming that 1 mol of MT (6600) binds 7 moles of Cd.

### GSH Assay

Total GSH (reduced + oxidized) was measured in the postmitochondrial fraction according to the method of (Tietze 1969) by using NWLSS Glutathione Assay Kit (Vancouver, WA). Briefly, an aliquot of the supernatant (50 μL) was deproteinized by adding 100 μL of an aqueous solution of 5 % metaphosphoric acid. After centrifugation, an aliquot (25 μL) of the supernatant was diluted by adding 500 μL of assay buffer. To 400 μL of this solution, 400 μL of assay buffer, 50 μL of 5,5′-dithiobis-2-nitrobenzoic acid (DTNB), and 50 μL of GSH reductase in assay buffer were added and incubated for 2 min 30 s at room temperature. Subsequently 50 μL of NADPH solution was added and the reduction rate of DTNB into 5-thio-2-nitrobenzoic acid (TNB) measured spectrophotometrically at 412 nm for 3 min. GSH was expressed as μmol/g wet weight.

### Lipid Peroxidation Assay

Lipid peroxidation was assessed by measuring malondialdehyde formation using the thiobarbituric acid (TBA) assay (Ohkawa et al. 1979). To 0.1 mL of the tissue homogenate, 0.2 mL of 8.1 % sodium dodecyl sulfate, 1.5 mL of 20 % acetic acid, 1.5 mL of 0.8 % TBA, and 0.6 mL of distilled water were added and vortexed. The reaction mixture was placed in a water bath at 95 °C for 1 h. After cooling, 1.0 mL of distilled water and 5.0 mL of butanol/pyridine mixture (15:1 v/v) were added and vortexed. After centrifugation, absorbance of the organic phase was determined at 532 nm. Tetraethoxypropane was used to prepare a calibration curve. The results were expressed as TBA-reacting substances (TBARS) (nmol/g wet weight).

### Statistical Analysis

Data are expressed as mean ± SD. Differences between the groups and subgroups were analyzed by one-way analysis of variance (ANOVA) followed by Duncan’s multiple range test (IBM SPSS Statistics 21, IBM Corporation, Somers, NY, USA). Differences at *P* < 0.05 were considered statistically significant.

## Results

Although enlarged liver and kidney are indicative of chronic Cd toxicity (Liu et al. 1998; Habeebu et al. 2000), in the present study subchronic dietary Cd (30 and 60 μg Cd/g) did not affect liver and kidney weights in wild and laboratory-bred bank voles (Table 1). In addition, no changes of hemoglobin and hematocrit values were observed on Cd exposure in the two groups of bank voles. Similarly, dietary Cd had no effect on body mass (Table 1), and food intake (0.16–0.18 g/g body weight/d) in these animals.

**Table 1:** Body and organ weights, hematological values, and incidence of histopathological changes in liver and kidney of male wild and laboratory-bred bank voles exposed to dietary Cd

Group	Final body mass (g)	Liver mass (g)	Kidney mass (g)	Hemoglobin (g/100 mL)	Hematocrit (%)	Histopathology	
						Liver	Kidney
Wild bank voles							
Control	15.5 ± 1.4	0.69 ± 0.08	0.17 ± 0.02	16.8 ± 1.3	48.0 ± 1.5	(−)	(−)
Cd-30	15.0 ± 1.5	0.68 ± 0.09	0.16 ± 0.02	16.0 ± 1.1	47.8 ± 2.0	(−)	(−)
Cd-60	15.2 ± 0.9	0.68 ± 0.07	0.16 ± 0.01	15.5 ± 0.9	46.0 ± 0.9	(+)	(+)
Laboratory-bred bank voles							
Control	15.0 ± 0.8	0.67 ± 0.08	0.16 ± 0.02	16.7 ± 1.3	48.3 ± 1.5	(−)	(−)
Cd-30	15.2 ± 1.1	0.68 ± 0.09	0.17 ± 0.02	15.9 ± 1.1	47.9 ± 2.0	(−)	(−)
Cd-60	15.3 ± 1.1	0.68 ± 0.08	0.17 ± 0.02	16.0 ± 1.2	47.0 ± 1.6	(−)	(−)

Values represent mean ± SD for *n* = 6. Bank voles received, for 4 weeks, control diet or diets containing 30 and 60 μg Cd/g. There were no statistically significant differences between the groups and subgroups. Histopathology: normal morphology (−), histopathological changes (+)

**Fig. 1:**
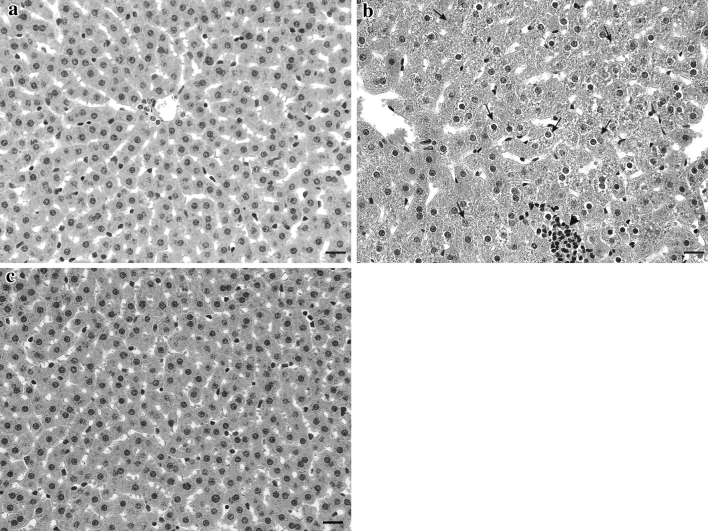
Representative photomicrographs of liver section from **a** control wild bank voles, **b** wild bank voles exposed to dietary Cd in quantity of 60 μg/g (hepatocyte swelling [*arrows*], leukocyte infiltration [*arrowhead*]), and **c** laboratory-bred bank voles exposed to dietary Cd in quantity of 60 μg/g (no damage is seen).* Scale bar* 20 μm

**Fig. 2:**
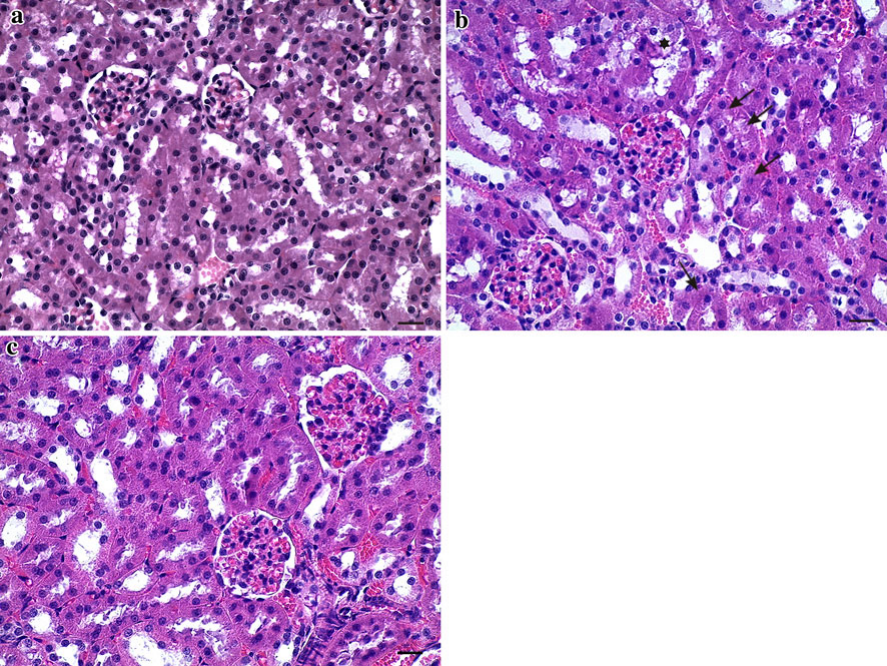
Representative photomicrographs of kidney section from **a** control wild bank voles, **b** wild bank voles exposed to dietary Cd in quantity of 60 μg/g (proximal tubule degeneration [*asterisk*], epithelial cell swelling [*arrows*]), and **c** laboratory-bred bank voles exposed to dietary Cd in quantity of 60 μg/g (no damage is seen).* Scale bar* 20 μm

The histopathological changes in the liver and kidneys are shown in Figures 1 and 2, respectively. Liver and kidney of control wild and laboratory-bred bank voles showed normal morphology (Figs. 1a and 2a). Normal morphology was also found in all laboratory-bred bank voles exposed to dietary Cd (Table 1). In contrast, focal hepatocyte swelling, vacuolation, and inflammation (leukocyte infiltration) (Fig. 1b) in liver—as well as focal proximal tubule degeneration, including epithelial cell swelling (Fig. 2b), in kidney—of all wild bank voles fed a diet containing 60 μg Cd/g was observed (Table 1).

Liver and kidney were also analyzed for Cd, MT, Zn, Cu, Fe, TBARS, and GSH contents (Tables 2 and 3). Accumulation of Cd in liver and kidney was dose-dependent (*P* < 0.0001) and reached similar levels (*P* *>* 0.1) in wild and laboratory-bred bank voles. In general, hepatic and renal MT levels followed a pattern similar to that of Cd accumulation: MT induction by Cd in laboratory-bred voles was not significantly greater (*P* > 0.1) than that in liver and kidney of wild animals. Subchronic consumption of Cd significantly increased (*P* < 0.01) Zn concentrations in liver and kidney to similar levels in wild and laboratory-bred bank voles, but hepatic and renal Cu was not affected by dietary Cd in the two groups of animals. In contrast, dietary Cd decreased (in a dose-dependent fashion) hepatic Fe in wild and laboratory-bred voles; however, decreased Fe was most pronounced in wild rodents exposed to dietary Cd at 60 μg/g. Renal Fe was not influenced by dietary Cd (*P* > 0.1) in all animals studied. Notably, lipid peroxidation, measured as TBARS in liver of wild bank voles fed a diet containing 60 μg Cd/g, decreased to 33 % of that observed in control animals. No changes of this process on Cd exposure were found in liver of laboratory-bred voles as well as kidney of rodents from the two groups. As listed in Table 2, dietary Cd did not significantly affect (*P* > 0.1) GSH concentrations in liver of wild and laboratory-bred bank voles. Renal GSH could not be detected by the method used in this study.

**Table 2:** MT, GSH and metal concentrations, and TBARS in liver of male wild and laboratory-bred bank voles exposed to dietary Cd

Group	Cd (μg/g wet weight)	MT (μg/g wet weight)	Zn (μg/g wet weight)	Cu (μg/g wet weight)	Fe (μg/g wet weight)	TBARS (nmol/g wet weight)	GSH (μmol/g wet weight)
Wild bank voles							
Control	0.14 ± 0.04^a^	4.95 ± 1.20^a^	23.0 ± 3.0^a^	3.5 ± 0.3^a^	260 ± 47^a^	113 ± 27^a^	6.6 ± 1.9^a^
Cd-30	5.92 ± 0.95^b^	50.5 ± 7.9^b^	25.4 ± 2.5^ab^	3.4 ± 0.3^a^	150 ± 40^b^	100 ± 15^a^	7.2 ± 2.1^a^
Cd-60	11.35 ± 1.81^c^	95.0 ± 13.0^c^	27.5 ± 3.5^b^	3.7 ± 0.4^a^	70 ± 20^c^	37 ± 10^b^	6.7 ± 2.5^a^
Laboratory-bred bank voles							
Control	0.13 ± 0.02^a^	5.87 ± 1.25^a^	23.5 ± 3.2^a^	3.9 ± 0.5^a^	350 ± 85^a^	108 ± 30^a^	6.9 ± 3.0^a^
Cd-30	5.84 ± 0.82^b^	62.0 ± 8.2^b^	25.8 ± 4.2^ab^	3.8 ± 0.4^a^	210 ± 47^b^	110 ± 15^a^	6.5 ± 2.1^a^
Cd-60	12.50 ± 1.65^c^	107 ± 20.0^c^	28.0 ± 3.6^b^	3.8 ± 0.6^a^	140 ± 20^b^	95 ± 20^a^	7.0 ± 2.2^a^

Values represent mean ± SD for *n* = 6. Bank voles received, for 4 weeks, control diet or diets containing 30 and 60 μg Cd/g. Means in the same column marked with a different superscript lower-case letter are significantly different (*P* < 0.05) (ANOVA and Duncan’s multiple range test)

**Table 3:** MT, metal concentrations, and TBARS in kidney of male wild and laboratory-bred bank voles exposed to dietary Cd

Group	Cd (μg/g wet weight)	MT (μg/g wet weight)	Zn (μg/g wet weight)	Cu (μg/g wet weight)	Fe (μg/g wet weight)	TBARS (nmol/g wet weight)
Wild bank voles						
Control	0.75 ± 0.20^a^	19.7 ± 4.6^a^	15.4 ± 2.2^a^	5.7 ± 0.9^a^	105 ± 20^a^	155 ± 30^a^
Cd-30	9.6 ± 1.3^b^	80 ± 11^b^	17.5 ± 3.0^ab^	5.5 ± 1.0^a^	110 ± 15^a^	157 ± 25^a^
Cd-60	18.0 ± 2.0^c^	150 ± 21^c^	22.2 ± 3.3^b^	5.6 ± 0.7^a^	104 ± 28^a^	148 ± 35^a^
Laboratory-bred bank voles						
Control	0.36 ± 0.15^a^	20.1 ± 4.2^a^	15.6 ± 2.0^a^	5.1 ± 0.5^a^	102 ± 21^a^	142 ± 18^a^
Cd-30	10.0 ± 1.5^b^	95 ± 13^b^	18.5 ± 3.0^ab^	5.6 ± 0.8^a^	105 ± 18^a^	150 ± 15^a^
Cd-60	18.5 ± 3.7^c^	171 ± 20^c^	23.0 ± 3.4^b^	5.4 ± 0.6^a^	95 ± 15^a^	148 ± 20^a^

Values represent mean ± SD for *n* = 6. Bank voles received, for 4 weeks, control diet or diets containing 30 and 60 μg Cd/g. Means in the same column marked with a different superscript lower-case letter are significantly different (*P* < 0.05) (ANOVA and Duncan’s multiple range test)

## Discussion

The results of the present study demonstrate dramatic differences in the development of Cd-induced liver and kidney injury between wild and laboratory-bred bank voles. Cd induced histopathological changes in liver and kidney only in wild animals despite the fact that tissue Cd accumulation was similar in the two groups of animals. These data indicate the following: (1) wild bank voles are more sensitive to Cd toxicity than those bred and raised under laboratory conditions; and (2) the difference in sensitivity is not related to total Cd accumulation.

The difference in susceptibility to Cd-induced liver and kidney injury between the two groups of bank voles could be related, however, to MT induction, GSH content, oxidative stress, and Zn, Cu, and Fe concentrations, which are considered to be critical factors during the development of Cd toxicity (Chan and Cherian 1992; Jacquillet et al. 2006; Jihen et al. 2008; Klaassen et al. 2009; Liu et al. 2009; Whittaker et al. 2011). Indeed, it has been shown that the difference in the susceptibility of rat strains to Cd-induced liver injury relates to the induction of MT (Kuester et al. 2002; Sabolić et al. 2010; Theocharis et al. 1994); still, other investigators have not demonstrated such a relation in mouse strains (Kershaw and Klaassen 1991). The present study showed that laboratory-bred bank voles treated with Cd produced nonsignificantly greater amounts of hepatic and renal MT than wild voles exhibiting histopathology. Furthermore, assuming that one mol of MT (6600) binds 7 moles of Cd, the Cd-binding capacity of hepatic and renal MT in wild animals was sufficiently high to potentially bind all Cd accumulated. Thus, MT does not appear to be the major factor responsible for the difference observed between the wild and laboratory-bred bank voles. Likewise, GSH, which is known to provide protection against Cd toxicity (Chan and Cherian 1992), could have had only a negligible effect because its content was relatively stable in all bank voles exposed to Cd (Table 2). Moreover, hepatic and renal Zn, which are also known to protect against Cd toxicity (Jacquillet et al. 2006; Jihen et al. 2008), increased to the same level in the two groups (Tables 2 and 3). This suggests that the fraction of Zn, which most likely was sequestered by MT, was ineffective in protecting against toxicity. It also seems unlikely that liver and kidney injury from Cd exposure in wild bank voles was due to increased oxidative stress (thought to be a cellular mechanism of toxicity [Thévenod and Friedmann 1999; Liu et al. 2009; Wang et al. 2010]) because hepatic and renal lipid peroxidation—as well as concentrations of pro-oxidant elements, such as Fe and Cu (Whittaker et al. 2011)—were similar or even lower than those in the laboratory-bred animals. Therefore, another mechanism is probably involved in Cd-induced liver and kidney injury in wild bank voles.

Numerous studies have shown that Cd decreases intestinal Fe absorption and its concentration in the liver of various animals, including the bank vole (Groten et al. 1991; Włostowski et al. 2000, 2003; Świergosz-Kowalewska and Holewa 2007). In the present study, dietary Cd brought about, in a dose-dependent pattern, an Fe decrease in liver of wild and laboratory-bred bank voles; however, the decrease in wild bank voles appeared to be more pronounced than that in the laboratory-bred animals (Table 2). Because Fe is an essential component of the Fenton reaction (hydroxyl radical production) as well as of several proteins and enzymes composing the mitochondrial respiratory chain, one would expect major changes to occur concurrently in the related oxidative processes, such as lipid peroxidation. Indeed, a dramatic decrease in lipid peroxidation in the liver of wild voles exhibiting the lowest Fe concentration (Table 2) suggests that this may be the case. Furthermore, the considerable decrease in hepatic Fe could lead to some disturbances in the mitochondrial respiration and ATP synthesis. If so, ATP deprivation could cause the loss of ionic control and cellular disintegration: The presence of hepatocyte swelling (Fig. 1b) in wild bank voles exposed to the highest dose of dietary Cd might support this assumption. In contrast to liver, kidney injury in wild bank voles was not accompanied by a decrease in Fe and lipid peroxidation (Table 3). This remains in some contrast to a previous study that showed a decrease in renal Fe and lipid peroxidation on Cd exposure (Włostowski et al. 2000); however, the discrepancy may be due to a different dietary Cd concentration and exposure time used in the two studies. Still, the occurrence of swollen proximal tubule epithelial cells (Fig. 2b) may also indicate dysfunction of mitochondrial respiration. This could be related to a Cd–MT complex released by the injured hepatocytes into the blood and taken up by the proximal tubule cells; notably, the complex has been shown to adversely affect mitochondrial respiration in these cells (Tang and Shaikh 2001). These data confirm the notion that dietary Cd may produce histopathological changes in liver and kidney indirectly through decreasing the hepatic Fe and Fe-dependent oxidative processes (Włostowski et al. 2000, 2003).

Although a significant decrease of hepatic Fe caused by dietary Cd in wild bank voles appears to be an important factor responsible for liver and kidney injury, the reason for the difference in the extent of decreased hepatic Fe in response to Cd exposure between wild and laboratory-bred bank voles remains unknown. It cannot be ruled out that the difference may be related to the fact that free-ranging bank voles, compared with those bred and raised in the laboratory, are subjected to various stressful conditions, including changes in food resources and ambient temperature, threats from predators, and interaction with conspecifics, which can elicit psychological stress in these animals (Marchlewska-Koj et al. 2003; Tidhar et al. 2007). Importantly, this stress has been shown to result in various pathophysiological processes, including impairment of Fe absorption, specifically the expression of ferroportin in enterocytes (Chen et al. 2009). Whether or not stress is responsible for the significant decrease of hepatic Fe on Cd exposure in wild bank voles remains to be elucidated in future studies.

In conclusion, these data indicate that free-ranging bank voles are more susceptible to Cd-induced liver and kidney injury than those bred and raised under laboratory conditions. The difference in sensitivity appears not to be related to Cd disposition, MT induction, GSH content, oxidative stress, or Zn concentration; however, it may be associated with a distinct decrease of hepatic Fe in response to Cd exposure between wild and laboratory-bred bank voles.
